# Structural and biochemical analyses of an aminoglycoside 2′-*N*-acetyltransferase from *Mycolicibacterium smegmatis*

**DOI:** 10.1038/s41598-020-78699-z

**Published:** 2020-12-09

**Authors:** Chang-Sook Jeong, Jisub Hwang, Hackwon Do, Sun-Shin Cha, Tae-Jin Oh, Hak Jun Kim, Hyun Ho Park, Jun Hyuck Lee

**Affiliations:** 1 Research Unit of Cryogenic Novel Material, Korea Polar Research Institute, Incheon, 21990 Republic of Korea; 2 Department of Polar Sciences, University of Science and Technology, Incheon, 21990 Republic of Korea; 3 Department of Chemistry and Nanoscience, Ewha Womans University, Seoul, 03760 Republic of Korea; 4 Department of Life Science and Biochemical Engineering, Graduate School, SunMoon University, Asan, 31460 Republic of Korea; 5 Genome-Based BioIT Convergence Institute, Asan, 31460 Republic of Korea; 6 Department of Pharmaceutical Engineering and Biotechnology, SunMoon University, Asan, 31460 Republic of Korea; 7 Department of Chemistry, Pukyong National University, 45 Yongso-ro, Busan, 48513 Republic of Korea; 8 College of Pharmacy, Chung-Ang University, Dongjak-gu, Seoul, 06974 Republic of Korea

**Keywords:** Structural biology, X-ray crystallography, Biochemistry, Biocatalysis, Enzyme mechanisms

## Abstract

The expression of aminoglycoside-modifying enzymes represents a survival strategy of antibiotic-resistant bacteria. Aminoglycoside 2′-*N*-acetyltransferase [AAC(2′)] neutralizes aminoglycoside drugs by acetylation of their 2′ amino groups in an acetyl coenzyme A (CoA)-dependent manner. To understand the structural features and molecular mechanism underlying AAC(2′) activity, we overexpressed, purified, and crystallized AAC(2′) from *Mycolicibacterium smegmatis* [AAC(2′)-Id] and determined the crystal structures of its apo-form and ternary complexes with CoA and four different aminoglycosides (gentamicin, sisomicin, neomycin, and paromomycin). These AAC(2′)-Id structures unraveled the binding modes of different aminoglycosides, explaining the broad substrate specificity of the enzyme. Comparative structural analysis showed that the α4-helix and β8–β9 loop region undergo major conformational changes upon CoA and substrate binding. Additionally, structural comparison between the present paromomycin-bound AAC(2′)-Id structure and the previously reported paromomycin-bound AAC(6′)-Ib and 30S ribosome structures revealed the structural features of paromomycin that are responsible for its antibiotic activity and AAC binding. Taken together, these results provide useful information for designing AAC(2′) inhibitors and for the chemical modification of aminoglycosides.

## Introduction

Aminoglycosides are broad-spectrum antibacterial compounds typically containing one aminocyclitol ring (the most common being 2-deoxystreptamine) linked to one or more amino sugars by glycosidic bonds. The primary target of aminoglycosides is the bacterial small ribosomal subunit. By binding to the 16S rRNA of the 30S ribosome at the tRNA acceptor aminoacyl site, aminoglycosides inhibit protein translation^1,2^.

Bacterial resistance mechanisms against aminoglycosides include mutation or methylation of certain 16S rRNA nucleotides involved in aminoglycoside binding, decreased aminoglycoside uptake, and chemical modifications of aminoglycosides by aminoglycoside-modifying enzymes. Among these mechanisms, enzyme-mediated chemical modifications of aminoglycosides are the most common and clinically relevant ones^3,4^. Three different classes of enzymes are well established: ATP-dependent phosphotransferases (*O*-phosphorylation, APH), ATP-dependent nucleotidyltransferases (*O*-nucleotidylation, ANT), and acetyl coenzyme A (CoA)-dependent *N*-acetyl-transferases (*N*-acetylation, AAC). There are four types of AACs—AAC(1), AAC(2′), AAC(3), and AAC(6′)—that acetylate the amino groups in aminoglycoside antibiotics using acetyl-CoA^5^. AAC(2′) enzymes generally promote the acetylation of dibekacin, gentamicin, kanamycin, netilmicin, and tobramycin. AAC(2′) was initially identified in *Providencia stuartii* [AAC(2′)-Ia], in which the presence of aminoglycosides led to the overexpression of this acetyltransferase^6^. Notably, other AAC(2′) enzymes have only been found in mycobacteria, including *Mycobacterium fortuitum* [AAC(2′)-Ib]^7^, *Mycobacterium tuberculosis* [AAC(2′)-Ic]^8,9^, *Mycobacterium smegmatis* [AAC(2′)-Id]^10^, and *Mycobacterium leprae* [AAC(2′)-Ie]. Until now, structures of AAC(2′) from *M. tuberculosis* (PDB codes 1M44, 1M4G, 1M4I, and 1M4D)^9^ and AAC(2′) from *P. stuartii* (PDB codes 5US1) have been determined^11^.

Here, we present the crystal structures of an AAC(2′) from *M. smegmatis* [AAC(2′)-Id] in its apo-form and AAC(2′)-Id complexed with CoA and different aminoglycosides (gentamicin, sisomicin, neomycin, and paromomycin). *M. smegmatis* is a nonpathogenic model organism representing other pathogenic *Mycobacterium* species; however, in some very rare cases, it may cause disease. Our biochemical analysis revealed that AAC(2′)-Id can bind to various aminoglycoside antibiotic substrates, with a strong preference for sisomicin. Structural analysis revealed the binding mode of four different substrates (gentamicin, sisomicin, neomycin, and paromomycin) to AAC(2′)-Id. Moreover, structural comparison between paromomycin-bound AAC structures and a previously determined paromomycin-bound 30S ribosome structure^2^ unveiled structural features of paromomycin that can be modified to design new paromomycin derivatives with reduced affinity for AAC enzymes. Collectively, these results may provide useful insights into the design of new antibiotics targeting antibiotic-resistant pathogens.

## Results and discussion

### Overall structure of AAC(2′)-Id

**Figure 1:**
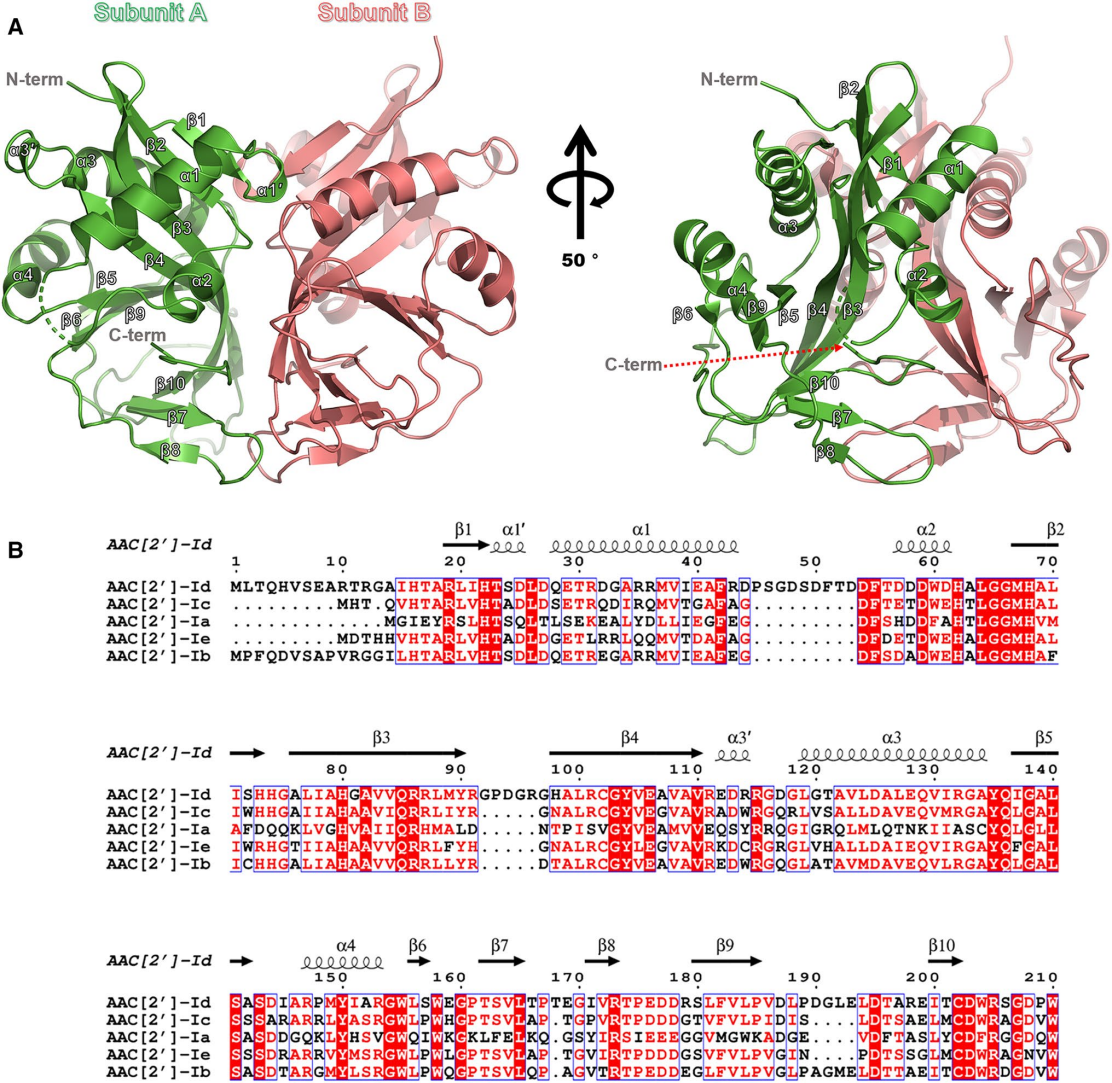
Crystal structure and multiple amino acid sequence alignment of AAC(2′)-Id. (**A**) The dimer structure of the apo-form of AAC(2′)-Id is shown as a cartoon. The right panel of the figure shows the same structure rotated by 50°. (**B**) Multiple amino acid sequence alignment of AAC(2′)-Id (UniProtKB code P94968) from *Mycolicibacterium smegmatis*, AAC(2′)-Ic from *Mycobacterium tuberculosis* (UniProtKB code P9WQG9), AAC(2′)-Ia from *Providencia stuartii* (UniProtKB code Q52424), AAC(2′)-Ie from *M. leprae* (UniProtKB code Q9CD24), and AAC(2′)-Ib from *M. fortuitum* (UniProtKB code Q49157).

The crystal structures of AAC(2′)-Id in its apo-form and CoA-bound form complexed with gentamicin, sisomicin, neomycin, and paromomycin have been determined at resolutions of 2.5, 2.17, 1.89, 2.0, and 2.05 Å, respectively (Table 1). The asymmetric unit of the apo-AAC(2′)-Id structure comprises a dimer of dimers. In contrast, the asymmetric units of the ternary antibiotic-CoA-AAC(2′)-Id complex structures contain only one dimer (Fig. 1A). Notably, the homodimer structure is similar in all crystal structures. Moreover, the dimerization of apo-AAC(2′)-Id was confirmed in solution via analytical ultracentrifugation (Supplementary Figure S1). The final model of apo-AAC(2′)-Id contains 742 amino acid residues (dimer of dimer, subunit A: 191 residues, subunit B: 180 residues, subunit C: 184 residues, subunit D: 187 residues), whereby the N-terminal loop region (residues 1–14) and the 45–51 loop region are commonly missing due to weak electron density in all subunit structures. The monomer structure of apo-AAC(2′)-Id has one long β-sheet surrounded by four α-helices. The dimerization of apo-AAC(2′)-Id is achieved by interactions among β1, α2–β2 loop, β3, β4, α3, β7–β8 loop, and β10 (Fig. 1B).

**Table 1:** X-ray diffraction data collection and refinement statistics.

Data set	Apo	Gentamicin + CoA	Sisomicin + CoA	Neomycin + CoA	Paromomycin + CoA
Data collection beamline	BL-5C, PAL	BL-5C, PAL	BL-5C, PAL	BL-5C, PAL	BL-7A, PAL
Space group	*P2*_*1*_*2*_*1*_*2*_*1*_	*P2*_*1*_*2*_*1*_*2*_*1*_	*P2*_*1*_*2*_*1*_*2*	*P2*_*1*_*2*_*1*_*2*	*P3*_*2*_
**Unit-cell**					
a, b, c (Å)	a = 69.0, b = 81.2, c = 129.7	a = 54.4, b = 60.2, c = 116.9	a = 77.8, b = 93.4, c = 61.3	a = 77.8, b = 94.9, c = 61.1	a = 56.3, b = 56.3, c = 122.8
α, β, γ (°)	α = β = γ = 90	α = β = γ = 90	α = β = γ = 90	α = β = γ = 90	α = β = 90, γ = 120
Wavelength (Å)	0.9794	1	1	1	0.97935
Resolution (Å)	50–2.5 (2.54–2.5)	50–2.17 (2.21–2.17)	50–1.89 (1.92–1.89)	50–2.0 (2.03–2.0)	50–2.05 (2.09–2.05)
Total reflections	173,968	116,530	236,552	349,488	153,759
Unique reflections	26,178 (1297)	20,679 (791)	36,656 (1755)	30,481 (1537)	27,416 (1389)
Average I/σ (I)	31.7 (5.4)	37.9 (4.4)	29.2 (2.3)	47.0 (8.6)	31.5 (10.4)
R_merge_	0.113 (0.475)	0.078 (0.325)	0.089 (0.525)	0.085 (0.341)	0.101 (0.266)
R_meas_	0.123 (0.513)	0.086 (0.363)	0.097 (0.582)	0.089 (0.356)	0.111 (0.293)
R_pim_	0.049 (0.192)	0.036 (0.159)	0.038 (0.247)	0.026 (0.100)	0.047 (0.123)
CC1/2	0.987 (0.918)	0.988 (0.997)	0.996 (0.877)	0.998 (0.983)	0.989 (0.957)
Redundancy	6.6 (7.1)	5.6 (4.9)	6.5 (5.3)	11.5 (12.5)	5.6 (5.7)
Completeness (%)	99.8 (100)	97.1 (76.5)	99.8 (96.5)	94.0 (96.7)	99.9 (100)
**Refinement**					
Resolution range (Å)	34.42–2.48 (2.57–2.48)	41.950–2.168 (2.246–2.168)	42.840–1.887 (1.954–1.887)	42.89–1.96 (2.03–1.96)	28.187–2.050 (2.123–2.050)
No. of reflections of working set	24,855 (2308)	19,624 (1855)	34,759 (3305)	28,833 (2484)	25,966 (2559)
No. of reflections of test set	1267 (134)	1005 (93)	1853 (162)	1522 (118)	1413 (178)
**No. of atoms**	5861	3107	3397	3356	3347
Protein	5705	2864	2915	2912	2893
Ligands	16	129	158	228	199
Solvent	140	114	324	216	255
R_work_	0.1930 (0.2429)	0.1987 (0.2404)	0.1861 (0.2271)	0.2053(0.2002)	0.1851 (0.1820)
R_free_	0.2694 (0.3625)	0.2459 (0.3323)	0.2272 (0.2579)	0.2541 (0.2527)	0.2359 (0.2594)
R.m.s. bond angle (°)	0.95	1.33	1.09	1.2	1.15
R.m.s. bond length (Å)	0.007	0.016	0.007	0.007	0.008
**Average B value (Å**^**2**^**)**	41.3	38.5	26.9	30.8	22.6
Protein	41.3	37.7	25.9	29.6	21.4
Ligands	53.4	57.1	31.6	43.5	34.4
Solvent	39.6	38.5	33.8	33.8	27.4
**Ramachandran plot**					
Favored (%)	93.18	96.19	98.1	97.84	96.45
Allowed (%)	5.59	3.54	1.63	1.89	3.01
Outliers (%)	1.23	0.27	0.27	0.27	0.55

Numbers in parentheses represent values in the highest resolution shell.

A structural homology search using the DALI server^12^ revealed that AAC(2′)-Id displays the highest structural similarity with AAC(2′) from *M. tuberculosis* [AAC(2′)-Ic, PDB code 1M44]^9^. In addition, AAC(2′) from *P. stuartii* [AAC(2′)-Ia, PDB code 5US1]^11^, acyl-CoA N-acyltransferase from *Pseudomonas aeruginosa* (PDB code 1XEB), and ElaA from *Escherichia coli* (PDB code 5Z6N) showed high structural similarity with AAC(2′)-Id (Table 2). It should be noted that so far there is no structural information on four ring containing-aminoglycoside (4,5-disubstituted aminoglycosides: neomycin and paromomycin) bound AAC(2′) structure. Thus, it is valuable to compare binding mode difference and enzyme kinetics of AAC(2′)-Id between three ring-containing aminoglycosides (4,6-disubstituted aminoglycosides: gentamicin and sisomicin) and four ring-containing aminoglycosides (neomycin and paromomycin).

**Table 2:** Results of the structural homology search using DALI search (DALI-Lite server).

Protein	PDB code	DALI Z-score	UniProtKB code	Sequence identity (%) with AAC(2′)-Id (aligned residue number)	References
Aminoglycoside 2′-*N*-acetyltransferase from *Mycobacterium tuberculosis*	1M44	29.3	P9WQG9	67 (177/177)	^9^
Aminoglycoside acetyltransferase from *Providencia stuartii*	5US1	25.1	Q52424	31 (176/177)	^11^
Acyl-CoA *N*-acyltransferase from *Pseudomonas aeruginosa*	1XEB	15.1	Q9I717	17 (138/148)	Not yet published
ElaA from *Escherichia coli*	5Z6N	15.1	P0AEH3	16 (140/148)	Not yet published

### Substrate specificity of AAC(2′)-Id

To determine the substrate specificity of AAC(2′)-Id, we performed steady-state kinetic parameter measurements using acetyl-CoA and various aminoglycoside substrates. The results showed that among the tested substrates, gentamicin exhibited the highest binding affinity for AAC(2′)-Id, with dissociation constants (K_m_) of (2.06 ± 0.36) × 10. However, regarding the specific activity (k_cat_/K_m_), tobramycin performed best due to its relatively high turnover rate constant (k_cat_ = 3.34 ± 0.10 s^−1^). Collectively, the kinetic results indicate that AAC(2′)-Id has a very broad substrate-binding specificity, similar to AAC(2′)-Ic^9^. AAC(2′)-Id was not active on streptomycin because the antibiotic does not contain 2′ amino groups in its chemical structure, indicating that AAC(2′)-Id exhibits strict regioselectivity.

### Substrate- and CoA-binding residues of AAC(2′)-Id

**Figure 2:**
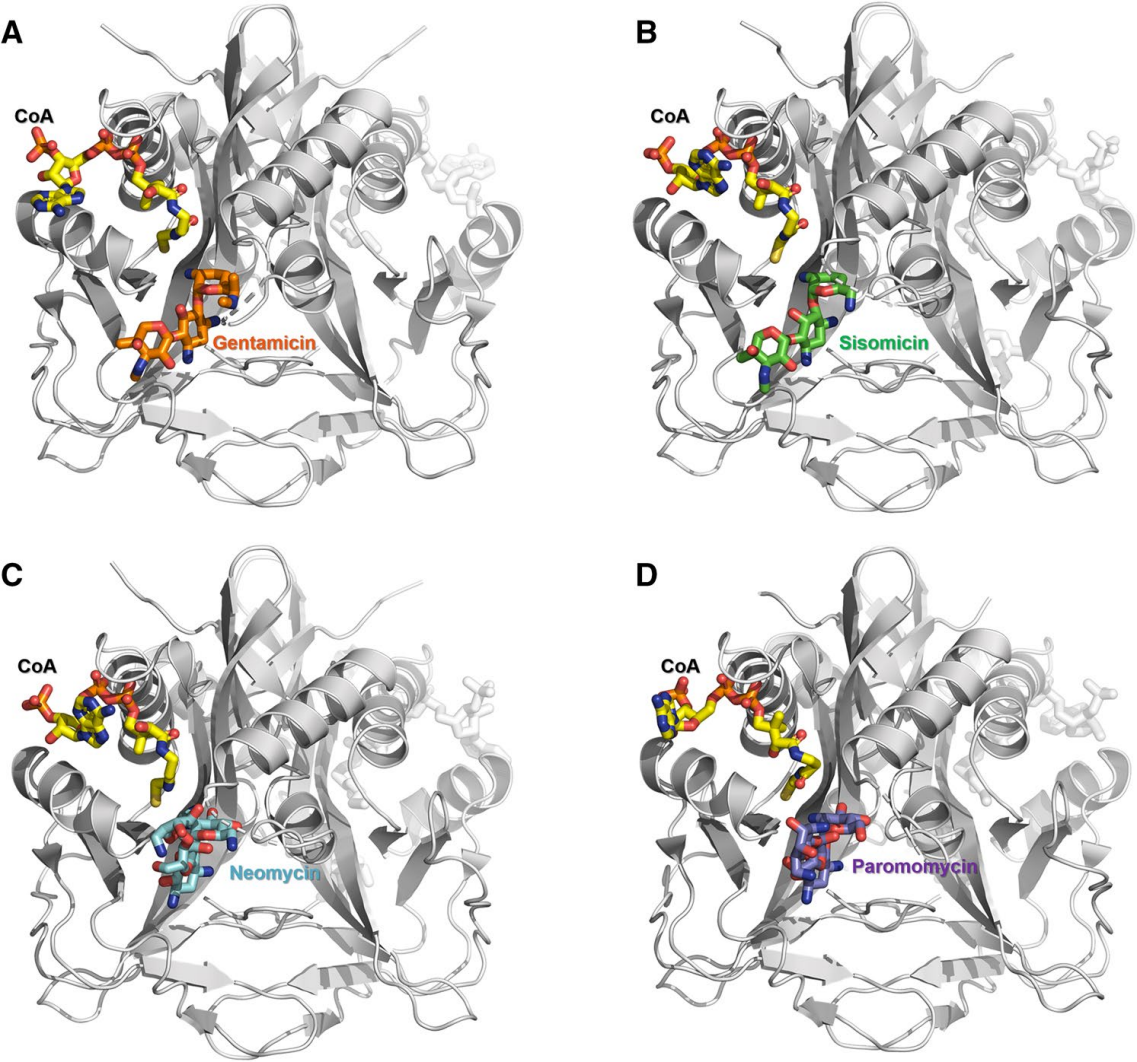
Structural comparison of the four aminoglycoside substrate-bound AAC(2′)-Id structures. (**A**) AAC(2′)-Id structure with bound CoA (yellow) and gentamicin (orange). (**B**) AAC(2′)-Id structure with bound CoA and sisomicin (green). (**C**) AAC(2′)-Id structure with bound CoA and neomycin (cyan). (**D**) AAC(2′)-Id structure with bound CoA and paromomycin (violet).

**Figure 3:**
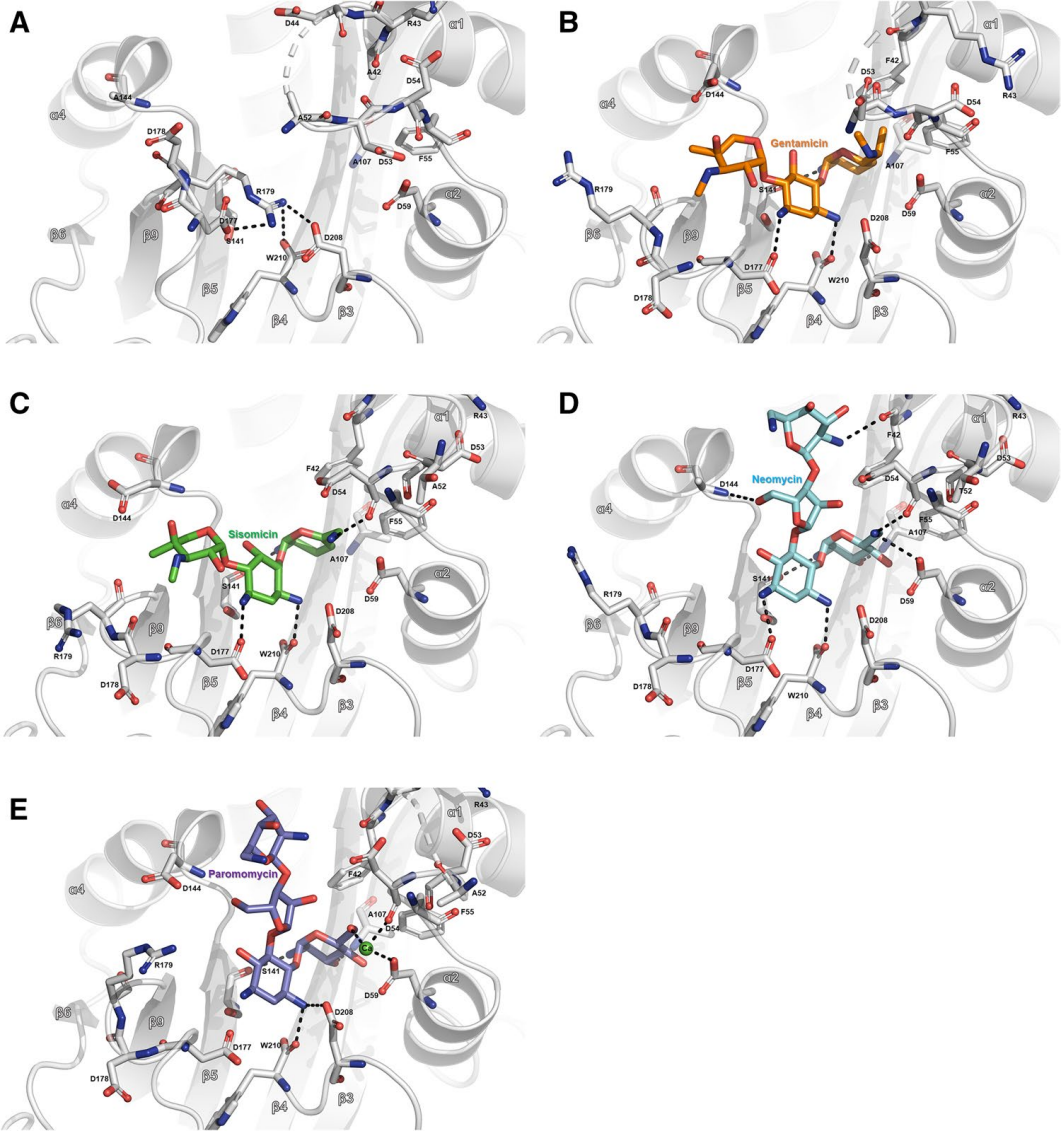
Differences in the interaction of AAC(2′)-Id with different substrates. (**A**) Close-up view of the substrate-binding site of apo-AAC(2′)-Id. (**B**) Close-up view of the substrate-binding site of gentamicin-bound AAC(2′)-Id. (**C**) Close-up view of the substrate-binding site of sisomicin-bound AAC(2′)-Id. (**D**) Close-up view of the substrate-binding site of neomycin-bound AAC(2′)-Id. (E) Close-up view of the substrate-binding site of paromomycin-bound AAC(2′)-Id.

To understand the substrate binding mechanism of AAC(2′)-Id, we determined the crystal structures of AAC(2′)-Id bound to four different aminoglycoside substrates (Fig. 2 and Supplementary Fig. S2). Structural comparison between the apo- and the substrate-CoA-bound AAC(2′)-Id structures revealed significant conformational differences in the α1–α2 loop, α4-helix, and β8–β9 loop region between the structures. The α1–α2 loop is the most flexible region in the structure of AAC(2′)-Id and thus misses some residues. This loop may move and cover the substrate-binding site in the apo-structure, whereas it exhibits a more open conformation in the substrate-bound AAC(2′)-Id structures. In addition, residue Arg179 located in the β8–β9 loop region also undergoes a dramatic conformational change upon substrate binding. In the apo-AAC(2′)-Id structure, Arg179 forms a salt bridge with Asp208 and Asp177, as well as hydrogen bonds with the carbonyl oxygen atom of Trp210. The salt bridge, however, is lost in all substrate-bound structures as Arg179 flips away from Asp208 and Asp177. Thus, the α1–α2 and β8–β9 loop regions may act as gatekeepers and thereby play a role in substrate recognition, substrate binding, product release, and solvent exclusion (Fig. 3).

**Figure 4:**
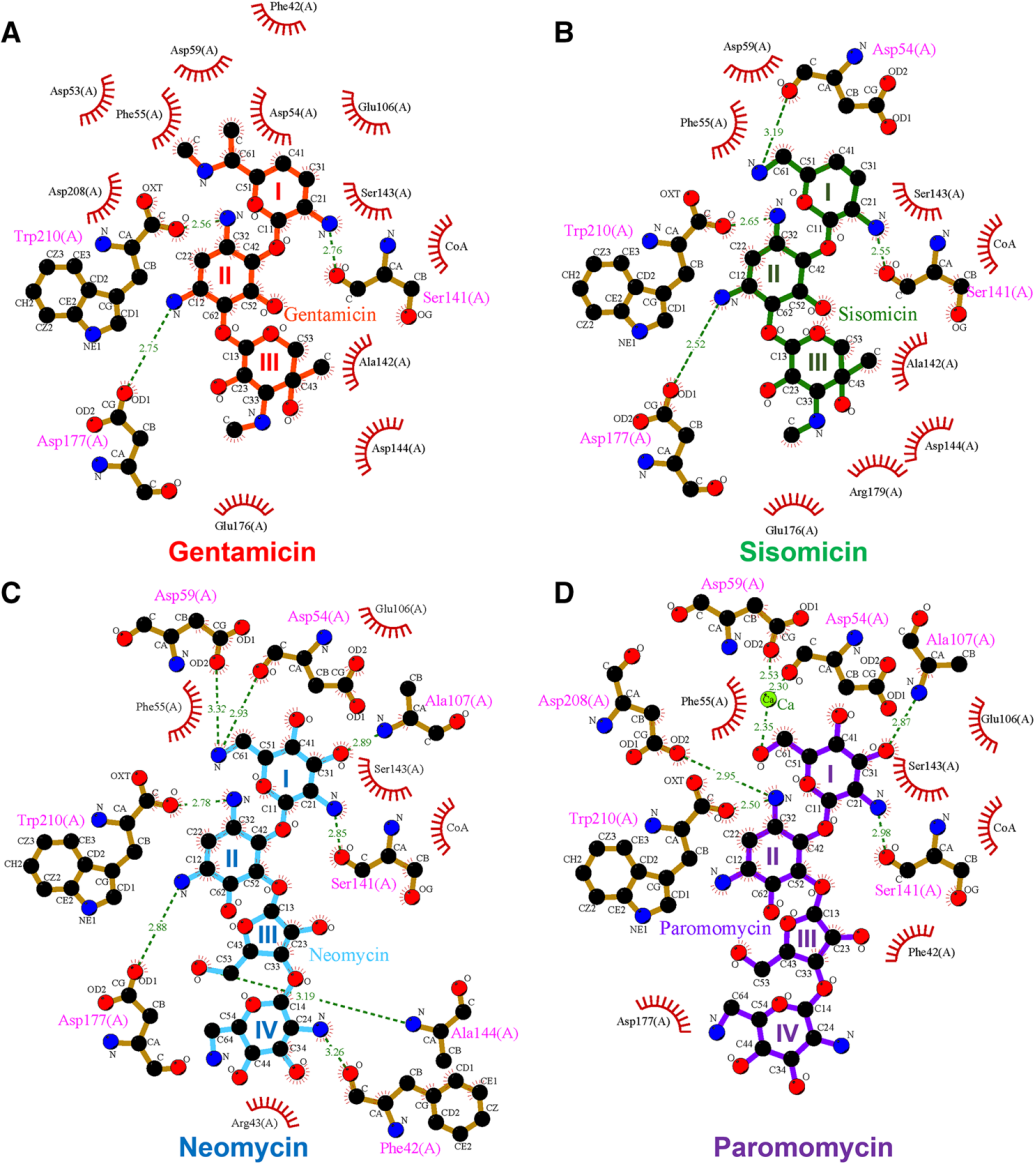
Overview of the substrate-AAC(2′)-Id interactions. Ligplot diagrams of (**A**) gentamicin, (**B**) sisomicin, (**C**) neomycin, and (**D**) paromomycin binding in AAC(2′)-Id. Hydrogen bonds are represented as green dashed lines, and hydrophobic interactions are shown as red arcs.

Gentamicin contains three ring structures referred to as the prime ring (ring I), central 2-deoxystreptamine core (ring II), and double prime ring (ring III). In the gentamicin-CoA-AAC(2′)-Id complex structure, the interactions between ring I of gentamicin and AAC(2′)-Id are hydrophobic, except for one hydrogen bond between Ser141 and the amino group of ring I (Fig. 4A). Ring II forms hydrogen bonds with Asp177 and Trp210, and ring III forms hydrophobic interactions with Ala142, Asp144, and Glu176. Sisomicin is structurally similar to gentamicin but has a unique unsaturated diamino sugar at the ring I position (Fig. 4B). In the sisomicin-CoA-AAC(2′)-Id complex structure, the overall interaction mode of sisomicin is identical to that of gentamicin, except for an additional hydrogen bond formed between the main chain carbonyl group of Asp54 and the second amino group on ring I of sisomicin (Fig. 4B). The chemical structures of both neomycin and paromomycin contain a different linkage at ring II and an additional hexose sugar ring (ring IV) compared with those of gentamicin and sisomicin. Paromomycin has a hydroxyl group at position 6′ of ring I, whereas neomycin bears an amino group at the corresponding position. In the neomycin-CoA-AAC(2′)-Id complex structure, the 6′-amino group of ring I forms direct hydrogen bonds with Asp54 and Asp59 (Fig. 4C). In contrast, the 6′-hydroxyl group of ring I of paromomycin exhibits calcium ion-mediated interactions with Asp54 and Asp59 (Fig. 4D). Compared to neomycin, paromomycin forms different interaction networks at the positions of oxygen atom of C61, leading to metal ion (calcium)-mediated interactions with the enzyme. This interaction may explain the higher binding affinity of paromomycin than neomycin in our activity assay. Taken together, the broad substrate specificity of AAC(2′)-Id could be explained by the fact that most of its interactions with aminoglycoside antibiotics are hydrophobic, except for several common hydrogen bonds and some flexible interactions of the α1–α2 loop, α4-helix, and β8–β9 loop region. In addition, metal ion-mediated interaction and side chain reorientations upon substrate binding could also contribute to the plasticity and broad substrate specificity of this enzyme (Fig. 4).

Structural comparison of the apo- and the four different aminoglycoside substrate-bound AAC(2′)-Id structures revealed that the ring II structure of each substrate exhibits common core interactions; the two nitrogen atoms of ring II form hydrogen bonds with Asp177 (or Asp208) and the carbonyl oxygen atom of Trp210. As described earlier, these negatively charged residues (Asp177 and Asp208) form a salt bridge with Arg179 in the apo-structure.

**Figure 5:**
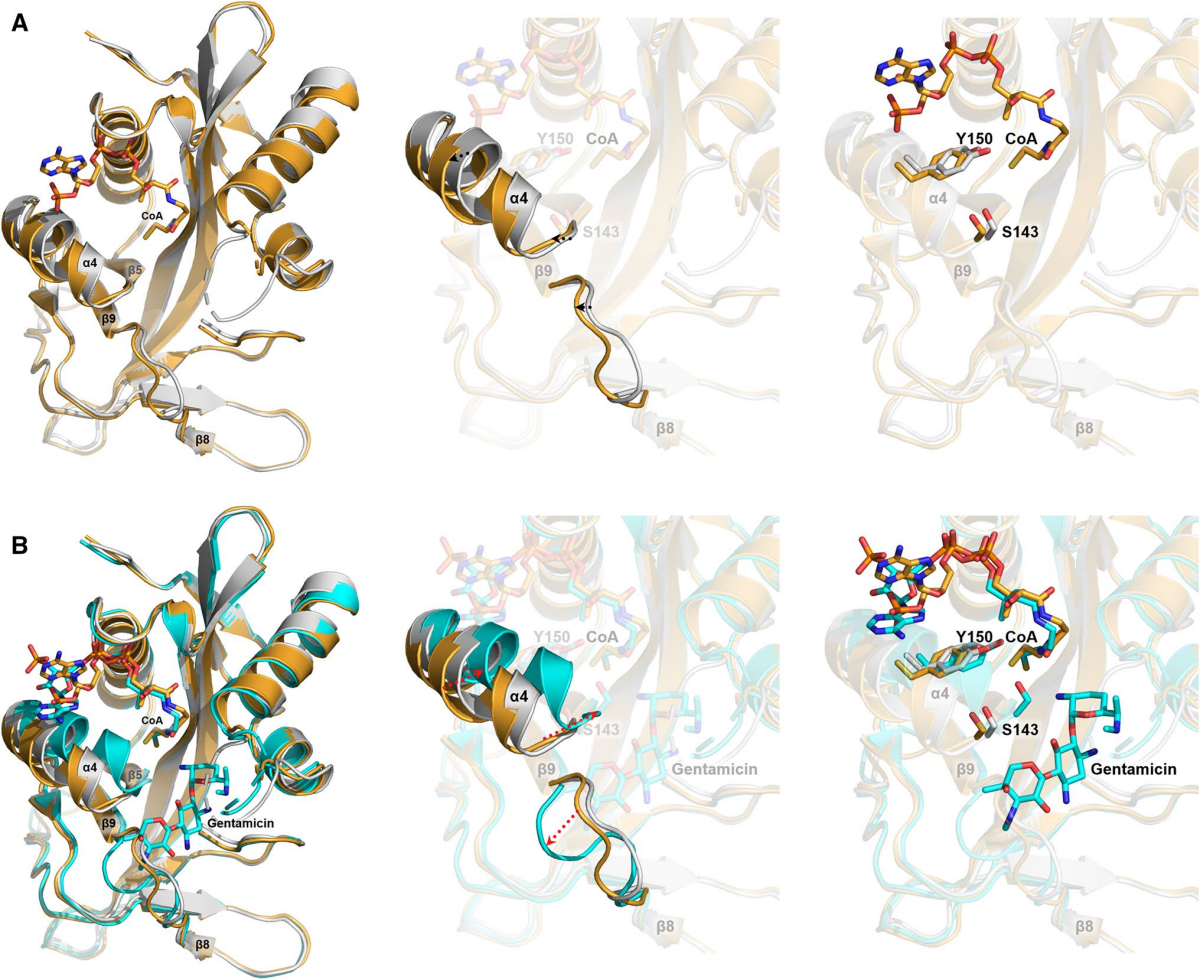
Structural comparison of the apo-form (gray), only CoA-bound form (orange), and CoA- and gentamicin-bound form (cyan) of AAC(2′)-Id. (**A**) Structural changes induced by CoA binding. CoA binding induces conformation changes in the α4-helix and β8–β9 loop region. (**B**) Structural changes induced by CoA and gentamicin binding. CoA and gentamicin binding induces a closing motion of the α4-helix and an opening motion of the β8–β9 loop region.

Chain A of the gentamicin-CoA-AAC(2′)-Id complex structure contains both a CoA and a gentamicin molecule, whereas chain B contains only a CoA molecule. Thus, the structural comparison of apo-AAC(2′)-Id with chain A and chain B of gentamicin-CoA-AAC(2′)-Id enabled us to identify structural changes induced by the sequential binding of CoA and gentamycin (Fig. 5). Structural superposition revealed that CoA binding induces only small conformational changes in the α4-helix region (Fig. 5A); however, subsequent binding of gentamicin causes more dramatic structural changes in the α4-helix and β8–β9 loop regions (Fig. 5B). Moreover, the binding of gentamicin leads to closer contact between CoA and the α4-helix, thus resulting in tighter binding of the coenzyme. In summary, the binding of CoA and gentamicin induces a closing motion of the α4-helix and an opening motion of the β8–β9 loop region. As the catalytically important residue Tyr150 and the catalytic residue Ser143 are located on the α4-helix and the β5–α4 loop, respectively, the structural change of the α4-helix observed here is closely related to the enzymatic reaction mechanism of AAC(2′)-Id. In our activity assay, the activity of the Tyr150Ala mutant was remarkably lower than that of wild-type AAC(2′)-Id (Table 3). Previous structural and activity assay studies on AAC(6′)-Ii showed that AAC(6′)-Ii also has a similar Tyr147 residue near the CoA binding site, similarly to the AAC(2′)-Id structure. The authors suggested that Tyr147 is important for making correct orientation between aminoglycoside substrates and CoA to transfer efficiently to the 6′-amino group of aminoglycosides^13^. It is also suspected that the Tyr150 residue in AAC(2′)-Id may have a similar function to Tyr147 in AAC(6′)-Ii.

**Table 3:** Steady-state kinetic parameters of AAC(2′)-Id for various substrates and acetyl-CoA.

Family	Coenzyme/substrate	*K*_*m*_ (μM)	*k*_*cat*_ (s^-1^)	*k*_*cat*_*/K*_*m*_ (M^−1^ s^−1^)
	Acetyl-CoA	(6.10 ± 1.20) × 10	0.34 ± 0.03	(5.67 ± 1.62) × 10^3^
Kanamycin	Amikacin	NA		
	Kanamycin	(1.67 ± 0.84) × 10^2^	0.13 ± 0.03	(8.17 ± 6.20) × 10^2^
	Tobramycin	(1.86 ± 0.10) × 10^2^	3.34 ± 0.10	(1.80 ± 0.16) × 10^4^
Gentamicin	Gentamicin	(2.06 ± 0.36) × 10	0.28 ± 0.00	(1.41 ± 0.29) × 10^4^
	Sisomicin	(7.30 ± 0.58) × 10	1.87 ± 0.08	(2.57 ± 0.41) × 10^4^
Neomycin	Neomycin	(7.76 ± 1.35) × 10	0.10 ± 0.00	(0.13 ± 0.03) × 10^4^
	Paromomycin	(1.04 ± 0.14) × 10^2^	1.08 ± 0.06	(1.04 ± 0.21) × 10^4^
Streptomycin	Streptomycin	NA		
**Tyr150Ala**				
	Acetyl-CoA	NA		
Kanamycin	Amikacin	NA		
	Kanamycin	NA		
	Tobramycin	NA		
Gentamicin	Gentamicin	NA		
	Sisomicin	(5.91 ± 1.06) × 10	0.01 ± 0.00	(2.18 ± 0.52) × 10^2^
Neomycin	Neomycin	NA		
	Paromomycin	NA		
Streptomycin	Streptomycin	NA		

*NA* no acetyl transfer activity was detected.

Notably, structural analysis of four different aminoglycoside substrate-bound AAC(2′)-Id structures revealed that the binding of all aminoglycosides to AAC(2′)-Id requires both α1–α2 and β8–β9 loop opening. However, three ring-containing aminoglycosides (4,6-disubstituted aminoglycosides: gentamicin and sisomicin) bind toward the β8–β9 loop region and make several interactions with this loop, whereas four ring-containing aminoglycosides (4,5-disubstituted aminoglycosides: neomycin and paromomycin) bind toward the α1–α2 loop region and make several interactions with it. Probably, this major binding mode difference between three ring-containing aminoglycosides and four ring-containing aminoglycosides results in the different binding affinity and catalytic efficiency among various aminoglycosides (Table 3). In our activity assay data, AAC(2′)-Id showed a stronger binding affinity and more catalytic efficiency to three ring-containing aminoglycosides (gentamicin and sisomicin) than four ring-containing aminoglycosides (neomycin and paromomycin). It is thought that three ring-containing-aminoglycosides form additional hydrophobic interactions with the β8–β9 loop as well as the β5–α4 loop. Thus, three ring-containing aminoglycosides may be able to bind to AAC(2′)-Id more favorably.

**Figure 6:**
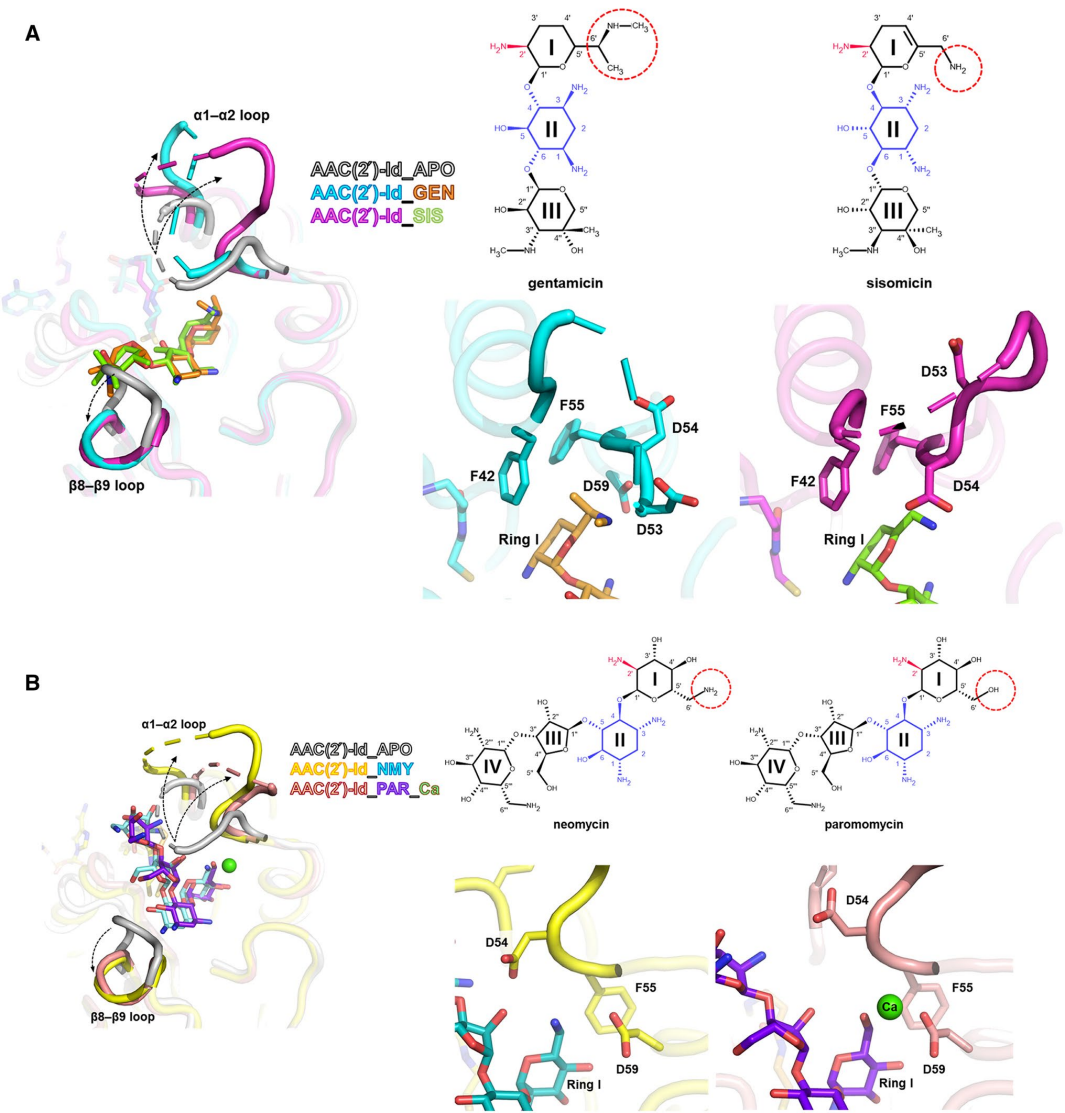
Comparison of substrate binding site and substrate binding mode of complex structures in AAC(2′)-Id. (**A**) Apo and three ring-containing aminoglycoside (gentamicin and sisomicin) complexed-AAC(2′)-Id structures are superimposed by Cα. Structure with gentamicin (orange) is colored in cyan, while the structure with sisomicin (green) is colored in magenta. (**B**) Apo and four ring-containing aminoglycoside (neomycin and paromomycin) complexed-AAC(2′)-Id structures are superimposed by Cα. Structure with neomycin (cyan) is colored in yellow, while the structure with paromomycin (purple) is colored in salmon. Close-up views of complex structures with corresponding substrates are represented with the same color code. The structural differences between three ring-containing aminoglycosides and four ring-containing aminoglycosides are marked with a red dot circle on each substrate structure.

Comparison analysis between gentamicin and sisomicin complex structures indicated that interaction between gentamicin and AAC(2′)-Id is stronger than that between AAC(2′)-Id and sisomicin. Unlike sisomicin, gentamicin contains methyl groups connected to the 6′ and amine group in ring I. The methyl group of 6′ forms a hydrophobic interaction with F42 and F55 (Fig. 6), and the other methyl group connected to the amine group interacts with the D59 backbone and probably influence the shift of the 45–53 loop region toward gentamicin and cover the active site. Consistent with this observation, the turnover rate of AAC(2′)-Id for gentamicin is slower than that for sisomicin. Paromomycin has the same chemical structure as neomycin, except that an oxygen atom is linked to C61 of paromomycin, while a nitrogen atom is linked to C61 of neomycin. The nitrogen atom linked to C61 of neomycin makes direct hydrogen bonds with the side chain OD2 of Asp59 and the backbone carbonyl group of Asp54 residue, whereas the oxygen atom linked to C61 of paromomycin forms metal ion interaction. This difference in interaction might explain why AAC(2′)-Id has ten times higher catalytic efficiency for paromomycin than for neomycin.

### Structural comparison of AAC(2′)-Id with other AAC enzymes

**Figure 7:**
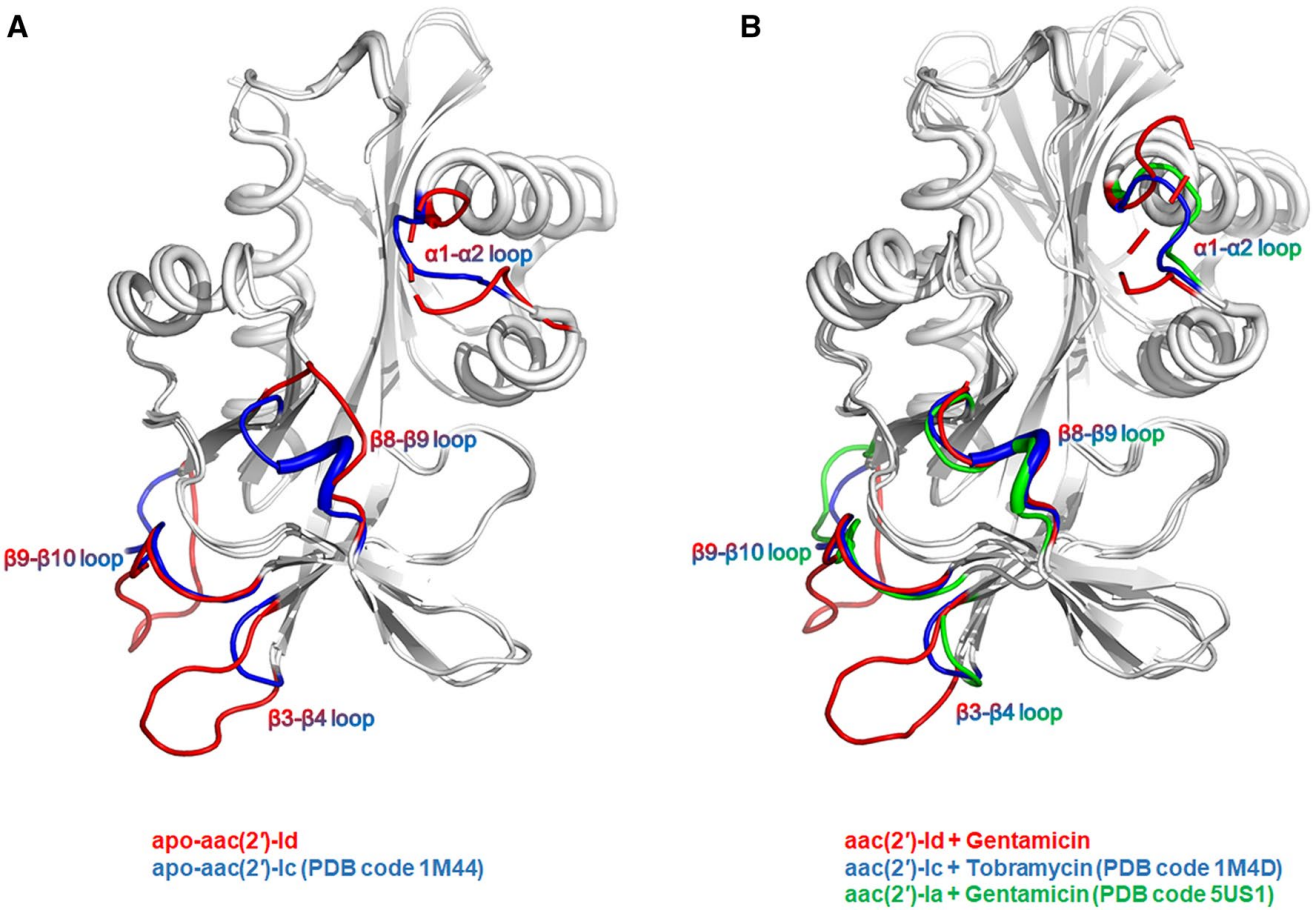
Structural comparison of AAC(2′)-Id, AAC(2′)-Ic, and AAC(2′)-Ia. (**A**) Loops that structurally differ between apo-AAC(2′)-Id and apo-AAC(2′)-Ic are highlighted in different colors. (**B**) Aminoglycoside and CoA binding induces remarkable conformational changes in the α1–α2 and β8–β9 loop regions of AAC(2′) enzymes.

Comparison of the structures of AAC(2′)-Id, AAC(2′)-Ic^9^, and AAC(2′)-Ia^11^ revealed remarkable structural and sequential differences in the α1–α2, β3–β4, β8–β9, and β9–β19 loop regions between these enzymes. The α1–α2 and β8–β9 loop regions are located near the active site and speculated to be involved in the initial substrate binding of each enzyme. Thus, the difference in substrate specificity between the examined AAC(2′) enzymes may be due to structural and amino acid residue differences in the α1–α2 and β8–β9 loop regions (Fig. 7).

**Figure 8:**
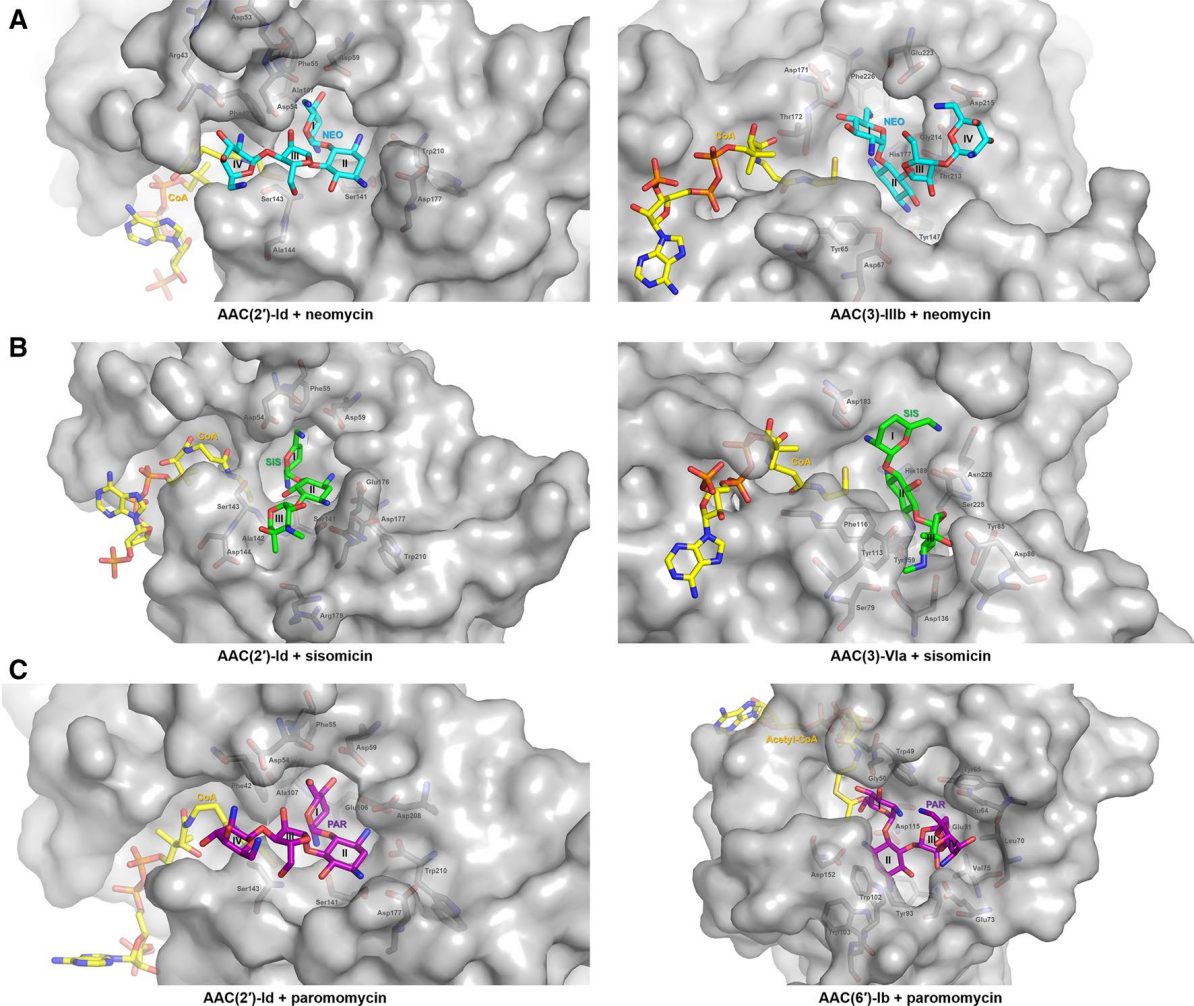
Regioselectivity mechanism of AAC enzymes. (**A**) Ring I of neomycin is most deeply buried in the active site of AAC(2′)-Id, whereas the rings II and III of neomycin exhibit intensive interactions with the active site of AAC(3)-IIIb (PDB code 6MB9). (**B**) Ring I of sisomicin is most deeply buried in the active site of AAC(2′)-Id, whereas the rings II and III of sisomicin exhibit intensive interactions with the active site of AAC(3)-VIa (PDB code 6BC3). (**C**) Ring I of paromomycin is most deeply buried in the active site of AAC(2′)-Id, whereas the rings II and III of paromomycin exhibit intensive interactions with the active site of AAC(6′)-Ib (PDB code 2VQY).

Next, the regioselectivity mechanism of AAC enzymes was investigated by structural comparison of the sisomicin- and CoA-bound AAC(3)-VIa (PDB code 6BC3)^14^, neomycin- and CoA-bound AAC(3)-IIIb (PDB code 6MB9)^15^, and paromomycin- and acetyl-CoA-bound AAC(6′)-Ib structures (PDB code 2VQY)^16^. Sequence identities among AAC enzymes are generally low (~ 10%); however, they all contain an acetyl-CoA and an aminoglycoside binding site. Structural comparison showed that aminoglycoside substrates bind with different conformations to each class of AACs, exhibiting different positions and rotation angles. The transfer of the acetyl group from acetyl-CoA to the aminoglycoside substrate requires optimal interaction distances and planar orientation of the substrate. For example, the binding mode of neomycin differs in AAC(2′)-Id and AAC(3)-IIIb. In the neomycin-bound AAC(2′)-Id structure, ring I of neomycin is most deeply buried in the enzyme's active site, whereas ring II is the most buried region in neomycin-bound AAC(3)-IIIb structure. In the sisomicin-bound AAC(3)-VIa structure, rings II and III of the antibiotic are the major interaction regions. In the case of paromomycin, rings I and II make multiple interactions with AAC(2′)-Id, while rings III and IV exhibit only a few interactions with the enzyme. However, paromomycin adopts a folded, L-shaped conformation in the structure of AAC(6′)-Ib, and all rings (I, II, III, and IV) exhibit strong interactions with the enzyme (Fig. 8).

**Figure 9:**
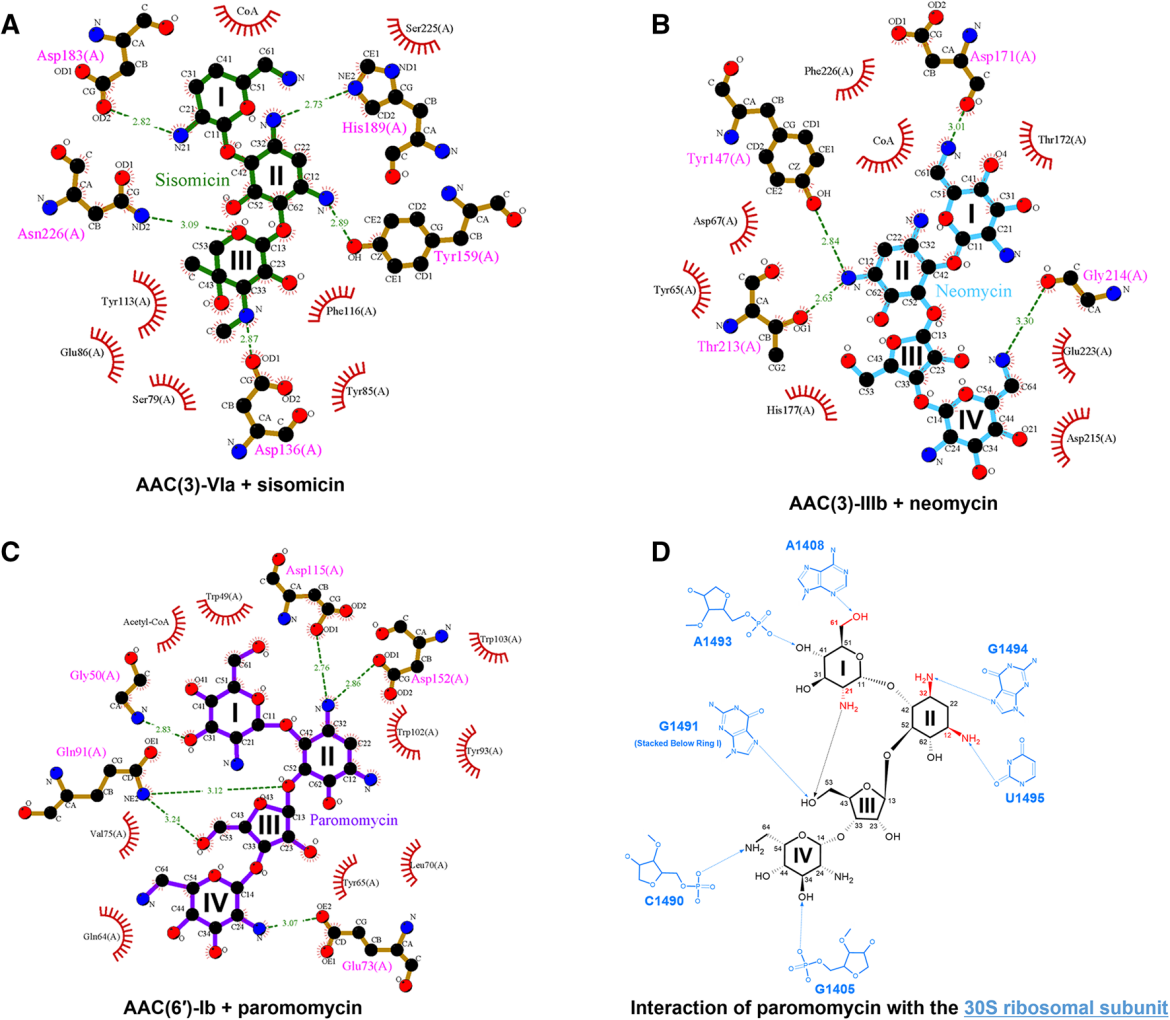
Overview of the interactions between different substrates and various AAC enzymes and those between paromomycin and the 30S ribosome. (**A**) Ligplot diagram of sisomicin (green) binding in AAC(3)-VIa. (**B**) Ligplot diagram of neomycin (blue) binding in AAC(3)-IIIb. (**C**) Ligplot diagram of paromomycin (purple) binding in AAC(6′)-Ib. Hydrogen bonds are represented as green dashed lines, and hydrophobic contacts are shown as red arcs. (**D**) The interactions between paromomycin and the 30S ribosome from *Thermus thermophiles* (PDB code 1FJG). The acetylation positions of the AAC enzymes are indicated in red.

Aminoglycosides bind to the bacterial 30S ribosome and thereby inhibit protein synthesis. Previous studies have described the crystal structures of the 30S subunit complexed with the antibiotics paromomycin, streptomycin, and spectinomycin^2^. Thus, it is worthwhile to investigate the interaction modes of paromomycin with the 30S ribosome and AAC enzymes. The present work shows that paromomycin exhibits different binding modes, depending on the protein to which it binds. These results provide novel directions in the design of chemically modified variants of paromomycin that exhibit antibiotic activity and overcome antibiotic resistance mediated by AAC enzymes. In detail, the O31 oxygen atom of ring I and the O23 oxygen atom of ring III are essential for the direct interaction between paromomycin and AAC enzymes. These oxygen atoms are, however, not involved in the binding of paromomycin to the 30S ribosome (Fig. 9). Therefore, chemical modifications of these oxygen atoms may inhibit the binding of paromomycin to AAC enzymes, while preserving that to the 30S ribosome for antibiotic activity.

In this study, we presented the first crystal structures of AAC(2′)-Id in its apo-form and AAC(2′)-Id complexed with coenzyme A and different aminoglycosides (neomycin, paromomycin, sisomicin, and gentamicin). Structural comparison of these structures enabled us to identify important residues for substrate binding and specificity. In addition, the present structures, together with previously solved aminoglycoside-bound structures (AAC enzymes and 30S ribosome), provide essential information for the design of novel AAC inhibitors and aminoglycoside derivatives. Previous studies have also shown that several aminoglycoside derivatives show increased activity against pathogens^11,17,18^.

## Methods

### Protein expression and purification

The open reading frame of the *aac(2′)-Id* gene from *M. smegmatis* with a length of 630 base pairs was retrieved from the database of the National Center for Biotechnology Information (accession no. WP_011726942). The *aac(2′)-Id* gene codon was optimized according to codon usage bias in *E. coli*. The sequence was synthesized, subcloned into the NdeI and XhoI sites of pET-28a(+) (Novagen, Madison, WI, USA), and transformed into *E. coli* BL21(DE3). The cells were grown to an OD_600_ of approximately 0.7 in Luria–Bertani medium containing 50 µg/mL kanamycin at 310 K, and expression was induced by 0.5 mM isopropyl β-d-1-thiogalactopyranoside. After 24 h of induction at 298 K, the cells were harvested by centrifugation and resuspended in 20 mM Tris–HCl, pH 8.5. The cells were disrupted by sonication, and the cell debris was removed by centrifugation at 20,000 × *g* for 40 min at 277 K. The resulting supernatant was loaded onto a nickel-nitrilotriacetic acid (Ni–NTA) column (TransGen Biotech). The column was equilibrated with a buffer consisting of 20 mM Tris–HCl (pH 8.5) and 30 mM imidazole. AAC(2′)-Id was eluted with the same buffer containing 300 mM imidazole. The protein fractions were pooled and loaded onto a column packed with 20 mL Q-Sepharose resin (GE Healthcare). The enzyme was then eluted with a gradient of 0‒1.0 M NaCl in 20 mM Tris–HCl, pH 8.5. The eluted fractions containing AAC(2′)-Id were pooled, concentrated, and subsequently treated with thrombin to cleave the N-terminal His-tag. Finally, thrombin and trace amounts of other contaminants were removed via size exclusion chromatography using a Superdex 200 column (GE Healthcare) pre-equilibrated with a buffer consisting of 20 mM Tris–HCl (pH 8.5) and 200 mM NaCl (buffer A). The purified AAC(2′)-Id was concentrated to approximately 17.8 mg/mL in buffer A using a spin column (Amicon Ultra Centrifugal Filter).

### Analytical ultracentrifugation

To investigate the oligomeric state of AAC(2′)-Id in solution, we performed analytical ultracentrifugation using a ProteomeLab XL-A (Beckman Coulter, Brea, CA, USA). Ultracentrifugation was performed in 20 mM Tris–HCl (pH 8.0) and 150 mM NaCl at 293 K. The sample was centrifuged at 40,000 rpm for 10 min, and the sedimentation profile was monitored at a wavelength of 280 nm. Data were analyzed using the SEDFIT program^19^.

### Crystallization and data collection

The AAC(2′)-Id apo-enzyme and the ternary complexes were crystallized using the sitting-drop vapor diffusion method in MRC crystallization plates (Molecular Dimensions) at 295 K. Screening for initial crystallization conditions was performed using commercially available sparse-matrix screens, including the MCSG I-IV (Molecular Dimensions), Index, and SaltRx screens (Hampton Research). Each crystallization drop was dispensed by a mosquito crystallization robot (TTP Labtech). Crystallization drops composed of 0.3 μL protein solution with an equal volume of reservoir solution were equilibrated against 70 μL reservoir solution. Crystals of the apo-enzyme were grown in 0.2 M ammonium acetate, 0.1 M Bis–Tris (pH 6.5), and 20% (w/v) PEG 3350. Crystals of the ternary complexes were obtained by complexing AAC(2′)-Id with 20 mM aminoglycoside and 5 mM CoA. Crystals of the ternary complex neomycin-CoA-AAC(2′)-Id were grown in 0.2 M potassium acetate and 20% (w/v) PEG 3350. Paromomycin-CoA-AAC(2′)-Id crystals were grown in 0.2 M calcium acetate hydrate, 0.1 M HEPES (pH 7.5), and 10% (w/v) PEG 8000. Sisomicin-CoA-AAC(2′)-Id was crystallized in 0.2 M sodium chloride, 0.1 M Tris (pH 8.5), and 25% (w/v) PEG 3350. Crystals of gentamicin-CoA-AAC(2′)-Id were crystallized in 0.1 M sodium citrate: citric acid (pH 5.5) and 20% (w/v) PEG 3000. For data collection, crystals were mounted in a loop and directly flash-frozen in a 100 K nitrogen stream after a quick soak in a cryoprotectant solution consisting of the respective reservoir solution supplemented with 20% glycerol.

X-ray diffraction data of all crystals were collected on the beamlines 5C and 7A at the Pohang Accelerator Laboratory (PAL, Pohang, Republic of Korea) using a CCD EIGER 9 M detector (Dectris, Baden, Switzerland) and a Quantum 270 CCD detector (ADSC, USA), respectively^20^. All data were indexed, integrated, and scaled using HKL2000^21^. Crystallographic data statistics are summarized in Table 1.

### Structure determination and refinement

The apo-form structure of AAC(2′)-Id was solved by molecular replacement with *MOLREP* from the *CCP4i* suite^22^ using the apo-AAC(2′)-Ic structure (PDB code 1M44) as the search model. The ternary complex structures were solved by molecular replacement using the apo-AAC(2′)-Id structure as the search model. Manual model building was performed in *WinCoot*^23^, and each model was refined using REFMAC5^24^ and *Phenix*^25^. All structures were deposited in the PDB (accession codes 7CRM, 7CS1, 7CS0, 7CSI, and 7CSJ for the apo-, gentamicin-, sisomicin-, neomycin-, and paromomycin-bound structures, respectively). All figures were prepared with *PyMOL*^26^.

### Enzyme activity assay

Kinetic assays were performed on AAC(2′)-Id using acetyl-CoA as the coenzyme, eight different aminoglycosides as substrates, and 5,5-dithiobis(2-nitrobenzoic acid) (DTNB) as an indicator. All assays were performed in 96-well plates with a final reaction volume of 200 μL at 25 °C. The reaction mixtures contained 50 mM MES (pH 6.5), 300 μM acetyl-CoA, 200 μM DTNB, and varying concentrations of aminoglycoside. The reaction was initiated by the addition of the enzyme, and the initial acetyltransferase rate of acetyl-CoA was determined by an increase in the absorbance at 412 nm, using an extinction coefficient (TNB) of 14,150 M^−1^ cm^−1^. The Michaelis–Menten parameters were obtained by fitting the initial velocity data using *OriginLab* software (version 8.0, Northampton, MA, USA).

## Supplementary Information

Supplementary Information.

## Data Availability

Atomic coordinates and structure factors have been deposited in the PDB under the accession codes 7CRM (apo-structure), 7CS1 (neomycin-bound structure), 7CS0 (paromomycin-bound structure), 7CSI (sisomicin-bound structure), and 7CSJ (gentamicin-bound structure).
