# Supplementary material for: Structural and biochemical analyses of an aminoglycoside 2′-N-acetyltransferase from Mycolicibacterium smegmatis
Source: Sci Rep. 2020 Dec 9;10:21503. doi: 10.1038/s41598-020-78699-z (PMC7725843; doi:10.1038/s41598-020-78699-z)
Supplement: Supplementary file 1 — Supplementary Information. [file 41598_2020_78699_MOESM1_ESM.docx]

**Supplementary Information for**

**Structural and biochemical analyses of an aminoglycoside 2'-N-acetyltransferase from *Mycolicibacterium smegmatis***

**Chang-Sook Jeong^1,2^, Jisub Hwang^1,2^, Hackwon Do^1^, Sun-Shin Cha^3^, Tae-Jin Oh^4,5,6^, Hak Jun Kim^7^, Hyun Ho Park^8,*^ & Jun Hyuck Lee^1,2,*^**

^1^Unit of Research for Practical Application, Korea Polar Research Institute, Incheon 21990, Republic of Korea; ^2^Department of Polar Sciences, University of Science and Technology, Incheon 21990, Republic of Korea; ^3^Department of Chemistry & Nanoscience, Ewha Womans University, Seoul 03760, Republic of Korea; ^4^Department of Life Science and Biochemical Engineering, Graduate School, SunMoon University, Asan, 31460, Republic of Korea; ^5^Genome-based BioIT Convergence Institute, Asan, 31460, Republic of Korea; ^6^Department of Pharmaceutical Engineering and Biotechnology, SunMoon University, Asan, 31460, Republic of Korea; ^7^Department of Chemistry, Pukyong National University, 45 Yongso-ro, Busan 48513 Republic of Korea; ^8^College of Pharmacy, Chung-Ang University, Dongjak-gu, Seoul 06974, Republic of Korea

*To whom correspondence should be addressed:

Prof. Hyun Ho Park, College of Pharmacy, Chung-Ang University, Dongjak-gu, Seoul 06974, Republic of Korea; Tel: +82-2-820-5930; Fax: +82-2-820-3033; Email: xrayleox@cau.ac.kr

Dr. Jun Hyuck Lee, Unit of Research for Practical Application, Korea Polar Research Institute, Incheon 21990, Republic of Korea; Tel: +82-32-760-5555; Fax: +82-32-760-5509; E-mail: junhyucklee@kopri.re.kr.

**Supplementary Figure S1.** Purification, crystallization, and X-ray diffraction data collection of AAC(2')-Id. (A) The purity of the AAC(2')-Id protein was confirmed with a 15% SDS-PAGE gel. (B) Analytical ultracentrifugation profiles of the AAC(2')-Id protein indicate that the protein exists as a dimer in solution. (C) Rod-shaped crystals of apo-AAC(2')-Id appeared after two days under the following crystallization conditions: 0.2 M Ammonium acetate, 0.1 M Bis-Tris (pH 6.5), and 20% (w/v) PEG 3350. The approximate dimensions of the crystals were 0.1 × 0.1 × 0.3 mm. (D) Representative X-ray diffraction pattern of an apo-AAC(2')-Id crystal.

**Supplementary Figure S2.** Unbiased |Fo|-|Fc| omit map of CoA and ligands from AAC(2′)-Id complexes. (A) Unbiased |Fo|-|Fc| Polder map of CoA (yellow) from CoA and sisomicin bound-AAC(2′)-Id structure. (B) Unbiased |Fo|-|Fc| Polder map of gentamicin (orange). (C) Unbiased |Fo|-|Fc| Polder map of sisomicin (green). (D) Unbiased |Fo|-|Fc| Polder map of neomycin (cyan). (E) Unbiased |Fo|-|Fc| Polder map of paromomycin (violet). F_O_-F_C_ Polder omit density (grey) is contoured at 3 σ.
